# Comparison of the Effects of Yokukansan and Yokukansankachimpihange on Glutamate Uptake by Cultured Astrocytes and Glutamate-Induced Excitotoxicity in Cultured PC12 Cells

**DOI:** 10.1155/2019/9139536

**Published:** 2019-05-27

**Authors:** Zenji Kawakami, Yuji Omiya, Kazushige Mizoguchi

**Affiliations:** Tsumura Kampo Research Laboratories, Kampo Research & Development Division, Tsumura & Co., 3586 Yoshiwara, Ami-machi, Inashiki-gun, Ibaraki 300-1192, Japan

## Abstract

The traditional Japanese Kampo medicine yokukansan (YKS) is effective for behavioral and psychological symptoms of dementia (BPSD) in patients with Alzheimer's disease. As the pharmacological mechanisms, YKS is known to protect astrocytes from thiamine-deficiency (TD)-induced decreased glutamate (Glu) uptake and neuron model cells (PC 12 cells) from Glu-induced death. Yokukansankachimpihange (YKSCH) is an alternative formula to YKS, in which Citrus unshiu peel and Pinellia tuber are added to the YKS components, and is sometimes used to treat BPSD, but its pharmacological properties remain unknown. This study aims to investigate the cellular pharmacological effects of YKS and YKSCH on glutamatergic pathways, compare their efficacy, and determine the differences and similarities in the activities between these formulations. First, we examined the effects of YKS and YKSCH on Glu uptake by cultured astrocytes under TD conditions. We observed significant ameliorative effects of YKS and YKSCH on the TD-induced decrease in Glu uptake, with a 50% effective dose of 8.9 ± 1.8 *μ*g/mL and 45.3 ± 9.2 *μ*g/mL, respectively. Second, using cultured PC12 cells as a model for neurons, we examined the effects of YKS and YKSCH on Glu-induced cell death. We observed that YKS and YKSCH had significant inhibitory effects on Glu-induced cell death, with a 30% effective dose of 51.4 ± 20.8 *μ*g/mL and 49.2 ± 11.0 *μ*g/mL, respectively. Thus, while YKSCH was less effective than YKS in ameliorating the TD-induced decrease in Glu uptake by astrocytes, the two drugs showed similar inhibitory effects on Glu-induced PC12 cell death. These findings are important for understanding the differences and similarities in pharmacological actions between these drugs.

## 1. Introduction

Composed of seven kinds of dried medicinal plants, yokukansan (YKS) is one of the traditional Japanese Kampo medicines. YKS has been approved by the Japanese Ministry of Health, Labor, and Welfare as a remedy for neurosis and insomnia and for irritability and night crying in children. YKS is reported to improve the behavioral and psychological symptoms of dementia (BPSD) such as delusions, hallucinations, and agitation/aggressiveness in patients with Alzheimer's disease, Lewy bodies, and other forms of senile dementia [1, 2]. These clinical findings are confirmed by two meta-analyses of randomized controlled trials [3, 4]. In animals, YKS is reported to ameliorate aggressive behaviors induced by thiamine-deficient (TD) [5, 6], zinc deficiency [7, 8], isolation stress [9], amyloid beta (A*β*) deposition in the brain [10], intracerebroventricular injection of A*β* oligomers [11], and glutamate (Glu) injection into the nucleus basalis of Meynert [12]. These observations are considered to reflect YKS effects on glutamatergic and serotonergic regulatory mechanisms. YKS was observed to attenuate excessive Glu release from presynaptic sites [7] and to improve reduced Glu uptake into astrocytes associated with Glu transporter dysfunction and Glu-induced neuronal death in cultures [13–15]. In the serotonergic system, for example, YKS exhibited partial agonistic actions for serotonin 1A (5-HT_1A_) receptors and upregulated these receptors [9, 16, 17]. YKS was also shown to downregulate 5-HT_2A_ receptor expression in the frontal cortical region of mice [18, 19].

Yokukansankachimpihange (YKSCH) is an alternative formula to YKS, containing an additional two medical herbs: Citrus unshiu peel (CUP) and Pinellia tuber (PT). In the theory of traditional medicines, CUP is thought to regulate qi (energy flow) and fortify the spleen and is used for the treatment of a variety of digestive dysfunctions including tympanites, nausea, vomiting, and dyspepsia [20, 21]. PT is also thought to dry dampness, and dispel phlegm, and is used to treat coughs with copious sputum [21, 22]. Although YKSCH is used for the same indications as YKS, the traditional uses of YKSCH differ somewhat. YKSCH is recommended for the treatment of patients with more severe loss of physical strength and more chronic conditions. Previous open-label trials showed that YKSCH improved BPSD such as delusions, irritability, and diurnal rhythm disturbance in patients with dementia [23–26]. A recent observational study showed that a combination of donepezil and YKSCH that contains Citrus reticulata instead of CPU significantly improved BPSD in patients with Alzheimer's disease, in which diurnal rhythm disturbance was significantly improved [27]. More recently, an open-label trial revealed that YKSCH tended to improve BPSD with significant improvement of apathy in Alzheimer's patients [28]. In animals, YKS and YKSCH produced the same degree of improvement in neuropsychiatric and gastrointestinal symptoms in TD rats [29]. In rats subjected to cholinergic degeneration in the nucleus basalis of Meynert, the ameliorative effect of YKSCH on aggressive behaviors is similar or somewhat weaker than that of YKS [12]. YKS but not YKSCH ameliorated the aggressive behavior of zinc-deficient mice housed individually [8, 30, 31]. Taken together, these clinical and basic research findings suggest that YKSCH improves BPSD and BPSD-like symptoms but with slightly less efficacy than that of YKS.

The present study aims to investigate the pharmacological effects of YKSCH and to compare its efficacy and action to that of YKS. For this purpose, we focused on the glutamatergic mechanisms involved in the ameliorative effects of YKS on aggressiveness. Then, we evaluated and compared the effects of YKS and YKSCH on TD-induced decreases in Glu uptake by astrocytes and Glu-induced excitotoxicity in cultured neuron-related PC12 cells.

## 2. Material and Methods

### 2.1. Drugs and Reagents

YKS and YKSCH were supplied by Tsumura & Co. (Tokyo, Japan). YKS is a dry powdered extract from a mixture of Atractylodes Lancea rhizome (4.0 g, rhizome of* Atractylodes Lancea* De Candolle), Poria sclerotium (4.0 g, sclerotium of* Poria cocos* Wolf), Cnidium rhizome (3.0 g, rhizome of* Cnidium officinale* Makino), Uncaria hook (3.0 g, thorn of* Uncaria rhynchophylla* Miquel), Japanese Angelica root (3.0 g, root of* Angelica acutiloba* Kitagawa), Bupleurum root (2.0g, root of* Bupleurum falcatum* Linné), and Glycyrrhiza (1.5 g, root and stolon of* Glycyrrhiza uralensis* Fisher). YKSCH comprises YKS with the two additional herbs, Pinellia tuber (PT; 5.0 g, tuber of* Pinellia ternate* Breitenbach) and Citrus unshiu peel (CUP; 3.0 g, peel of* Citrus unshiu* Markovich). Extract qualities were standardized based on the good manufacturing practice as defined by the Japanese Ministry of Health, Labour, and Welfare. The yields of YKS and YKSCH were 15.9 and 15.8%, respectively. In the present study, the concentration of YKSCH was set 1.4-fold higher than that of YKS in order to equalize the YKS amount included in both extracts.

**Figure 1:**
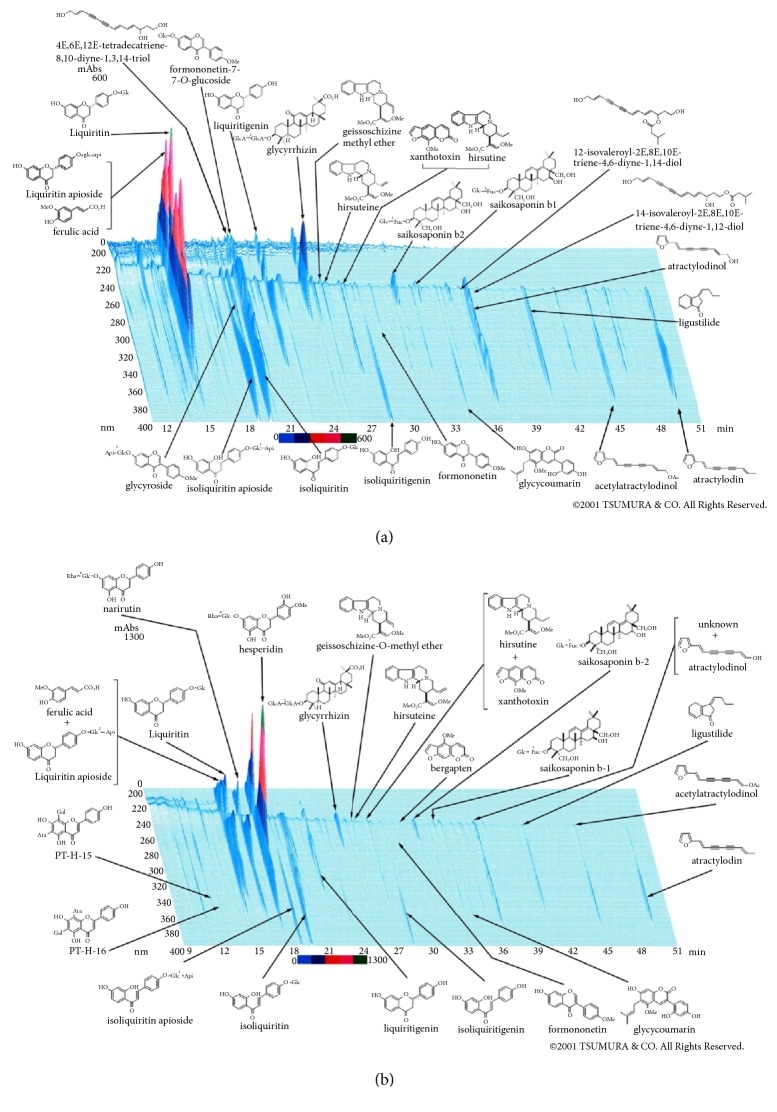
The three-dimensional HPLC profiles of YKS (a) and YKSCH (b).

The three-dimensional high-performance liquid chromatography (3D-HPLC) profiles of the representative batch of YKS or YKSCH are shown in Figure 1. For the analysis of components, the dried extract (1.0 g) of YKS or YKSCH was extracted with methanol (20 mL) under ultrasonication for 30 min and was centrifuged at 3000 rpm for 5 min. The supernatants were filtered with a membrane filter (0.45 *μ*m) and then submitted for HPLC analysis (30 *μ*L). HPLC apparatus consisted of a Shimadzu LC 10A (analysis system software: CLASS-M10A ver. 1.64, Tokyo, Japan) equipped with a multiple wavelength detector (UV 200-400 nm) (Shimadzu SPD-M10Avp, diode array detector), an auto injector (Shimadzu CTO-10AC). HPLC conditions were described as follows: column, ODS (TSK-GEL 80TS, 250 × 4.6 mm i.d., TOSOH, Tokyo, Japan); eluent, (A) 0.05M AcONH_4_ (pH 3.6) (B) 100% CH_3_CN. A linear gradient of 90% of A and 10% of B changing over 60 min to 0% A and 100% B was used. (And 100% B was continued for 20 min.); temperature, 40°C; flow rate, 1.0 mL/min. In the analysis, the maximum detection sensitivity of YKS was set at 600 milli-absorbance (mAbs), and that of YKSCH was set at 1300 mAbs because of the high content of a CUP-derived flavonoid, hesperidin.

The compounds shown on the chromatogram were classified on the basis of the constituent herbs of YKS and YKSCH (Table 1), in which the compound compositions identified from both formulas differed. This is considered to be the difference in the detection sensitivity. Another possible reason is the influence of the combination of specific crude drugs on the extraction efficiency. For example, the addition of PT to a certain formula has been reported to lower pH values in the extract solution, which reduces extraction efficiency of the compounds derived from Uncaria hook [32] or Glycyrrhiza [33, 34].

The reagents used in cell culture experiments, including DL-threo-*β*-hydroxy-aspartic acid (TBHA), dehydroquinate (DHK), pyrithiamine hydrobromide, Dulbecco's modified Eagle's medium (DMEM), DNase, glutamate dehydrogenase, *β*-nicotinamide adenine dinucleotide, 1-methoxyphenazine methosulphate, and Triton X-100, were purchased from Sigma-Aldrich (St. Louis, MO, USA). EDTA, HEPES, and 3-(4,5-dimethylthial)-2,5-diphenyltetrazalium bromide (MTT) were purchased from Dojindo (Kumamoto, Japan). Other chemicals were purchased from commercial sources.

### 2.2. Preparation of Primary Cultured Astrocytes

Primary cultured astrocytes were prepared according to the procedure described previously [35] with some modifications [13]. In brief, newborn-rat neopallia were mechanically disrupted by pipetting in DMEM/Ca- and Mg-free phosphate buffered saline [PBS(-)] (1:1). The suspension was filtered through a sterile nylon mesh with 100 *μ*m pores (Corning, Corning, NY, USA). The filtrate was passed through sterile lens cleaning paper (Fujifilm, Tokyo, Japan). Cells (3.75 × 10^6^) were seeded into a 75-cm^2^ culture flask (Corning) with DMEM containing 7.5 mM glucose, 2 mM glutamine, 25 mM NaHCO_3_, and 10% horse serum. The next day, the culture medium was replaced with DMEM containing 25 mM sorbitol, 2 mM glutamine, 25 mM NaHCO_3_, and 10% dialyzed horse serum, and the cells were incubated for 2 weeks. After the incubation period, the cultures were returned to the glucose-containing DMEM medium, and the purity of astrocytes in the culture was examined by immunocytochemical staining using an antibody to glial fibrillary acidic protein (GFAP), a specific marker for astrocytes (Supplementary Figure 1). We confirmed that at least 95% of the cells were astrocytes.

The high-purity cultured astrocytes were harvested from the substratum using Puck's solution (pH 7.2) containing 3 mM EDTA, 2 mM pyruvate, 7.5 mM glucose, 0.02% DNase, and 10 mM HEPES. Approximately 20 000 cells/cm^2^ were seeded into 96-well Primaria culture plates (Corning) and used in the following experiments after the cells became confluent.

### 2.3. Glu Uptake in Cultured Astrocytes

TD-subjected astrocytes were prepared according to the procedure described by Hazell [36] to induce TD as follows: confluent astrocytes were reseeded onto 96-well plates and cultured for 5 days in a custom-designed DMEM medium lacking in thiamine (CSTI, Sendai, Japan) and containing 5% horse serum in the presence of 10 *μ*M pyrithiamine, an inhibitor of the enzyme that produces the active form of thiamine. Control astrocytes were cultured for the same amount of time in DMEM medium including thiamine and containing 5% horse serum. TD astrocytes were treated with YKS or YKSCH as follows: astrocytes were cultured for 5 days in TD medium with various concentrations of YKS or YKSCH that had been filtered through a 0.22-*μ*m filter.

Glu uptake was evaluated in cells after culturing for 5 days. Thus, 100 *μ*M Glu was exogenously added to astrocyte cultures. After incubating for 5 h, an aliquot of the culture medium was carefully collected. The extracellular concentration of Glu was determined according to a colorimetric method described previously [37] with minor modifications. In brief, 50 *μ*l of the medium was mixed with an equal volume of substrate mixture containing 20 U/mL glutamate dehydrogenase, 2.5 mg/mL *β*-nicotinamide adenine dinucleotide, 0.25 mg/mL MTT, 100 *μ*M 1-methoxyphenazine methosulfate, and 0.1% Triton X-100 in 0.05 M Tris-HCl buffer (pH 8.2). The mixture was incubated at 37°C for 10 min, and the reaction was stopped by adding 100 *μ*L of a stop solution (pH 4.7) containing 50% dimethylformamide and 20% sodium dodecyl sulfate (SDS). The amount of formazan produced from MTT by Glu was colorimetrically determined at a test wavelength of 540 nm and a reference wavelength of 690 nm using a microplate reader. The Glu concentration of each sample was estimated from the standard curve constructed for each assay using cell-free medium containing known concentrations of L-glutamate.

The average Glu concentration of the TD-induced astrocyte (OD_TD_) sample was subtracted from the Glu concentration of the test-substance–treated sample (OD_test_) or the average Glu concentration of the control to calculate the extent of amelioration, as follows: Amelioration (%) = 100 − [(OD_test_–OD_TD_)/(OD_control_–OD_TD_)] × 100.

### 2.4. Glu-Induced PC12 Cell Death

PC12 cells were obtained from Dainippon Sumitomo Pharma (Osaka, Japan) and maintained at 37°C in 95% air and 5% CO_2_ with 95% relative humidity in RPMI 1640 medium (Thermo Fisher Scientific, Waltham, MA, USA) supplemented with 5% fetal calf serum, 10% heat-inactivated horse serum, penicillin (50 U/mL), and streptomycin (50 *μ*g/mL) until used in experiments.

On the day of the experiments, PC12 cells were seeded into 96-well microplates (5,000 cells/well in 100 *μ*L medium) in RPMI 1640 medium supplemented with 5% dialyzed fetal calf serum, 10% dialyzed horse serum, penicillin (50 U/mL), and streptomycin (50 *μ*g/mL) without phenol red. Forty-eight hours after seeding, the medium was replaced with one of three types of fresh culture medium (medium without Glu as a control, medium with 8 mM Glu, and medium with 8 mM Glu plus various concentrations of YKS or YKSCH) and the cells incubated for 24 h. Cell survival then was evaluated using the MTT reduction assay [38].

The MTT reduction assay was performed as follows. To each well of a 96-well plate was added 20 *μ*L of 5 mg/mL MTT dissolved in PBS(−), followed by incubation at 37°C for 5 h. The reaction was stopped by the addition of 100 *μ*L of solubilization solution (10% SDS in 0.01 N HCl). The blue formazan formed from MTT by the reaction was dissolved by additional incubation for 18 h at 37°C. The absorbance of the formazan solution was measured using an Infinite M200 microplate reader (Tecan, Grödig, Austria) at a test wavelength of 540 nm and a reference wavelength of 690 nm.

The average absorbance of the Glu-induced cell death (OD_Glu_) was subtracted from the absorbance of the test-substance–treated sample (OD_test_) or the average control absorbance (OD_control_) to calculate the % amelioration, as follows: Amelioration (%) = [(OD_test_–OD_Glu_)/(OD_control_–OD_Glu_)] × 100.

### 2.5. Statistical Analysis

Data are presented as the mean ± SEM. The statistical significance of differences between groups in cell culture experiments was assessed by Student's* t*-test or one-way analysis of variance (ANOVA) followed by Dunnett's post hoc test.* P *< 0.05 was considered significant.

## 3. Results

### 3.1. Effect of Drugs on TD-Induced Decrease in Glu Uptake by Astrocytes

**Figure 2:**
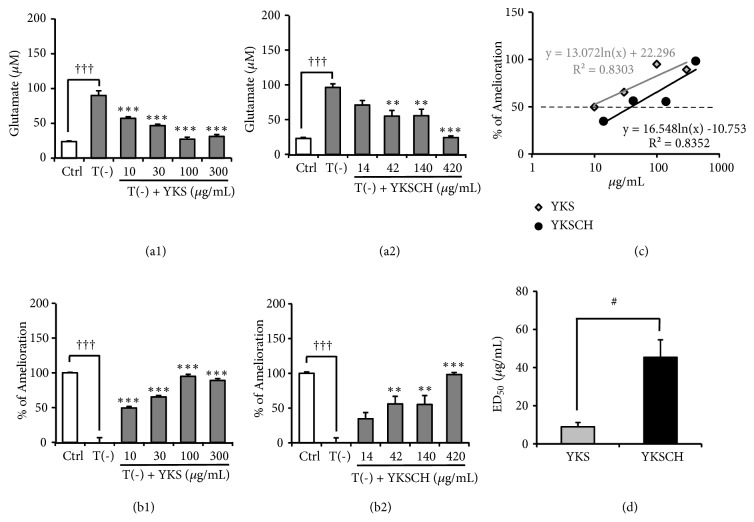
Effects of YKS or YKSCH on TD-induced decrease in Glu uptake by cultured astrocytes. Astrocytes were cultured for 5 days in (1) Ctrl: thiamine-containing normal medium, (2) T(−): TD medium, or (3) T(−) medium containing YKS (10–300 *μ*g/mL) or YKSCH (14–420 *μ*g/mL). (a1); (a2) Glu concentration of cultured Glu-containing medium (100 *μ*M) for 5 h. (b1); (b2) Amelioration rate as determined from Glu concentration. (c) 50% effective dose (ED_50_) as calculated from the regression equation for the amelioration rate. (d) Average ED_50_ compared between YKS and YKSCH. Data are presented as the mean ± SEM ((a1), (a2), (b1), and (b2): n = 6; (d): n = 5). ^†††^*P* < 0.001 vs. Ctr; *∗∗P* < 0.01 and *∗∗∗P* < 0.001 vs. T(−): one-way ANOVA + Dunnett's test. ^#^*P* < 0.05 vs. YKS: Student's* t*-test.

Under TD conditions, the extracellular Glu concentration remained approximately 100 *μ*M, the initial concentration of the medium, indicating that Glu uptake into TD astrocytes was almost completely inhibited. YKS and YKSCH treatment significantly decreased extracellular Glu in a concentration-dependent manner (*F*_(5,30)_ = 51.364,* P *< 0.001 and* F*_(5,30)_ = 20.747,* P *< 0.001, respectively) (Figures 2(a1) and 2(a2)), suggesting that both drugs ameliorated TD-induced impairment of glutamate uptake by astrocytes. The Glu concentration of the medium was converted to amelioration rate to allow comparison between the experiments (Figures 2(b1) and 2(b2)). The 50% effective dose (ED_50_) for amelioration was calculated using the regression equation for the amelioration rate (Figure 2(c)). Each experiment was repeated 5 times, and the average ED_50_ was calculated (average ED_50_: YKS, 8.9 ± 1.8 *μ*g/mL; YKSCH, 45.3 ± 9.2 *μ*g/mL). The ED_50_ differed significantly between YKS and YKSCH (*P* < 0.05) (Figure 2(d)).

**Figure 3:**
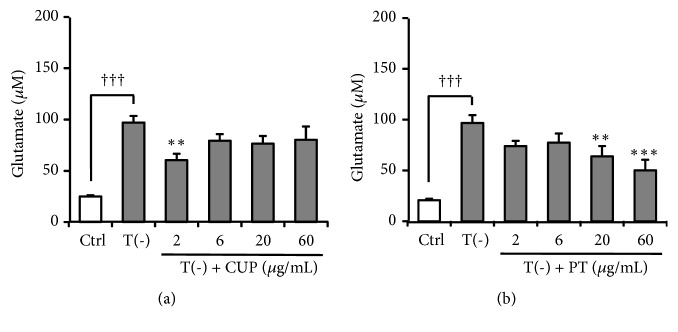
Effect of CUP or PT on TD-induced decrease in Glu uptake by cultured astrocytes. Astrocytes were cultured for 5 days in (1) Ctrl, thiamine-containing normal medium; (2) T(−), TD medium, or (3) T(−) medium containing CUP ((a) 2–60 *μ*g/mL) or PT ((b) 2–60 *μ*g/mL). Glu concentration of cultured Glu-containing medium (100 *μ*M) was evaluated after 5 h. Data are presented as the mean ± SEM (n = 6). ^†††^*P* < 0.001 vs. Ctrl; *∗∗P* < 0.01 and *∗∗∗P* < 0.001 vs. Glu: one-way ANOVA + Dunnett's test.

The inhibitory effects of CUP and PT, both of which are components of YKSCH, on TD-induced impairment of Glu uptake by astrocytes were examined. The TD-induced decrease in Glu uptake was ameliorated by PT (*F*_(5,30)_ = 10.744;* P *< 0.001) but not by CUP (*F*_(5,30)_ = 10.544;* P *< 0.001) (Figure 3).

**Figure 4:**
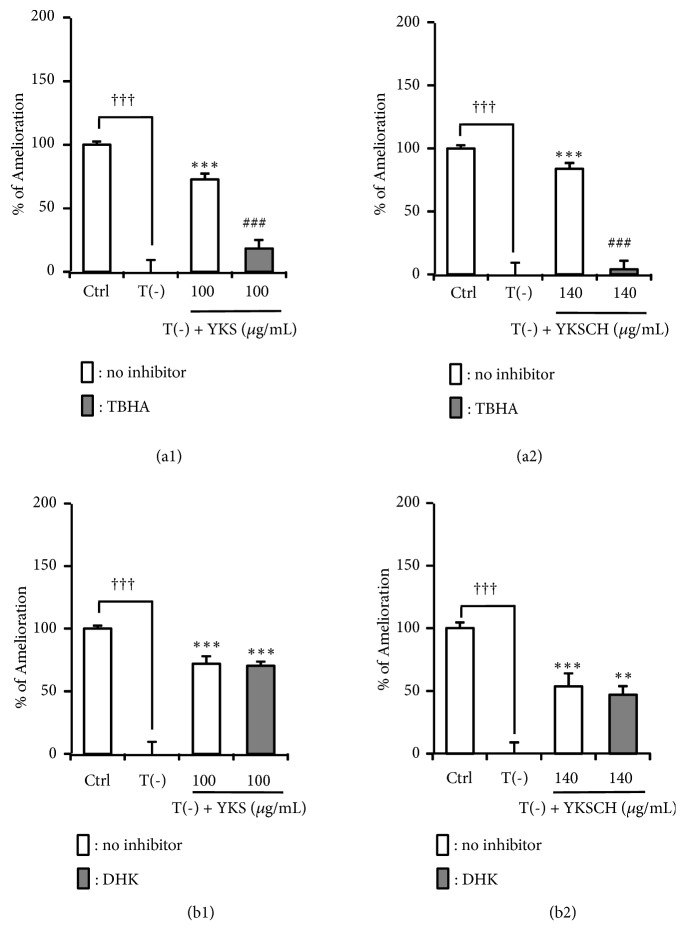
Effects of TBHA and DHK on amelioration of TD-induced impairment of Glu uptake by YKS and YKSCH. Astrocytes were cultured for 5 days in (1) Ctrl, thiamine-containing normal medium, (2) T(−), TD medium; or (3) T(−) medium containing YKS (100 *μ*g/mL) or YKSCH (140 *μ*g/mL). Extracellular Glu concentration was measured 5 h after addition of 100 *μ*M Glu with 300 *μ*M TBHA (a1, a2) or 1 mM DHK (b1, b2). Data are presented as the mean ± SEM (n = 6). ^†††^*P* < 0.001 vs. Ctrl, *∗∗P* < 0.01, *∗∗∗P* < 0.001 vs. T(−), and ^###^*P* < 0.001 vs. YKS or YKSCH: one-way ANOVA + Dunnett's test.

Figure 4 shows the effect of the Glu transporter inhibitors TBHA (nonspecific inhibitor of transporters including glutamate aspartate transporter [GLAST]) and DHK (specific inhibitor of glutamate transporter 1 [GLT-1]) on the amelioration of TD-induced decreased Glu uptake by YKS (100 *μ*g/mL) and YKSCH (140 *μ*g/mL). The decrease was significantly ameliorated by treatment with YKS or YKSCH, which was significantly blocked by TBHA (*F*_(3,20)_ = 61.701,* P *< 0.001 and* F*_(3,20)_ = 47.617,* P *< 0.001, respectively) but not DHK (*F*_(3,20)_ = 48.329,* P *< 0.001 and* F*_(3,20)_ = 26.505,* P *< 0.001, respectively) (Figure 4), suggesting that the amelioration by YKS and YKSCH predominantly occurs through effects on GLAST dysfunction under the present experimental conditions.

### 3.2. Effect of Drugs on Glu-Induced PC12 Cell Death

**Figure 5:**
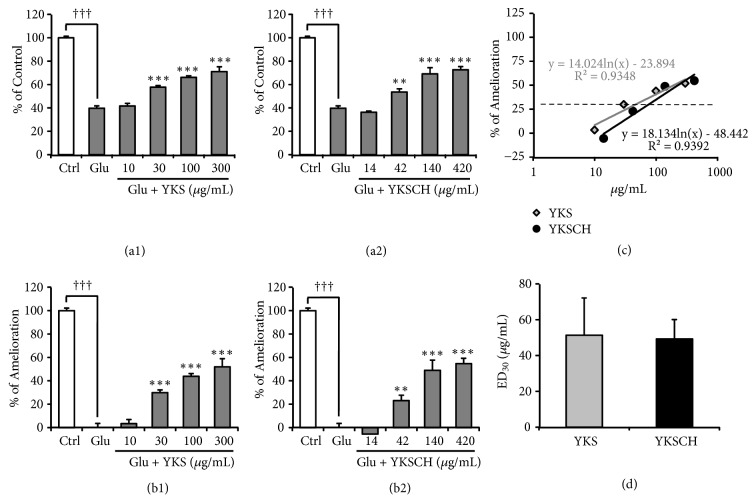
Effect of YKS or YKSCH on Glu-induced PC12 cell death. PC12 cells were incubated for 24 h in media containing 8 mM Glu + YKS (10–300 *μ*g/mL) or YKSCH (14–420 *μ*g/mL). The control medium (Ctrl) contained no Glu. (a1, a2) Survival rate calculated as %MTT activity relative to control PC12 cells (n = 6). (b1, b2) Amelioration rate as determined from survival rate. (c) 30% effective dose (ED_30_) as calculated from the regression equation for the amelioration rate. (d) Comparison of the average ED_30_ between YKS and YKSCH. Data are presented as the mean ± SEM ((a1), (a2), (b1), and (b2): n = 6; (d): n = 5). ^†††^*P* < 0.001 vs. Ctrl; *∗∗P* < 0.01, and *∗∗∗P* < 0.001 vs. Glu: one-way ANOVA + Dunnett's test.

The suppressive effects of YKS and YKSCH on Glu-induced PC12 cell death were evaluated by MTT assay, showing that these treatments prevented cell death in a concentration-dependent manner (*F*_(5,30)_ = 92.811,* P* < 0.001 and* F*_(5,30)_ = 67.363,* P* < 0.001, respectively) (Figures 5(a1) and 5(a2)). The cell survival rate was converted to the amelioration rate to allow comparison between experiments (Figures 5(b1) and 5(b2)). The 30% effective dose (ED_30_) for amelioration was calculated using the regression equation for the amelioration rate (Figure 5(c)). Each experiment was repeated 5 times, and the average ED_30_ was calculated. The average ED_30_ did not differ significantly between YKS (51.4 ± 20.8 *μ*g/mL) and YKSCH (49.2 ± 11.0 *μ*g/mL) (Figure 5(d)).

**Figure 6:**
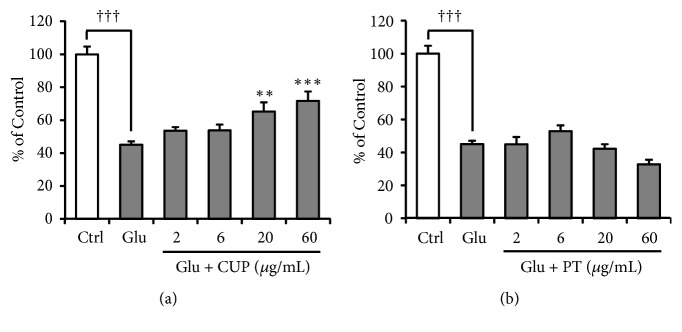
Effect of CUP or PT on Glu-induced PC12 cell death. PC12 cells were incubated for 24 h in medium containing 8 mM Glu + CUP ((a) 2–60 *μ*g/mL) or PT ((b) 2–60 *μ*g/mL). The control medium (Ctrl) contained no Glu. Survival rate calculated as the %MTT activity relative to control PC12 cells. Data are presented as the mean ± SEM (n = 6). ^†††^*P* < 0.001 vs. Ctrl; *∗∗P* < 0.01 and *∗∗∗P* < 0.001 vs. Glu: one-way ANOVA + Dunnett's test.

The inhibitory effects of CUP and PT on Glu-induced PC12 cell death were also examined. Glu-induced cell death was decreased by treatment with CUP (*F*_(5,30)_ = 21.289,* P *< 0.01;* F*_(5,30)_ = 47.660,* P *< 0.001) but not PT (Figure 6).

## 4. Discussion

This study presents two major findings. First, YKSCH facilitated the uptake of Glu into astrocytes subjected to TD, with weaker efficacy than that of YKS. Second, YKSCH inhibited Glu-induced excitotoxicity in PC12 cells, with efficacy similar to that of YKS.

Recent reviews estimate that among adults ≥ 65 years of age, half of the males and one-third of the females were at risk for inadequate thiamine intake [39, 40]. Thiamine is phosphorylated to thiamine diphosphate (TDP) by thiamine pyrophosphokinase. TDP is a cofactor for enzymes associated with glucose metabolism, including transketolase, pyruvate dehydrogenase, and *α*-ketoglutarate dehydrogenase. Decreased activity of these TDP-dependent enzymes has been reported in neurodegenerative diseases such as Alzheimer's and Parkinson's diseases [41]. Previous studies have demonstrated that BPSD-like behaviors such as anxiety, depression, muricide, attacking, and startle responses, as well as impaired learning and memory are observed in TD rats and mice [42, 43]. Collins [44] and Robertson [45] have demonstrated that astrocytes are among the first cells to be affected by TD in advance of neuronal death. Symptomatic TD is associated with increased extracellular Glu concentrations in focal regions of the brain [46, 47]. TD in cultured astrocytes resulted in decreased Glu uptake [36], caused by dysfunction of astrocyte Glu transporters [13]. In our previous study, we showed that YKS improved the decreased Glu uptake by cultured astrocytes under TD condition [13]. In the present study, effects of YKSCH as well as YKS were evaluated using the same system, and their efficacy was compared. As shown in Figure 2, the ameliorative effect of YKSCH on TD-induced decreased Glu uptake by astrocytes was weaker than that of YKS. This result suggests that the additional herbs in YKSCH (CUP and/or PT) might negatively affect Glu transport. However, unexpectedly, these herbs instead ameliorated the TD-induced decrease in Glu uptake (Figure 3). Another possible interpretation of this result is that CUP and/or PT might interfere with the ameliorative effects of YKS components when together in the formula of YKSCH. It should be noted that the ameliorative effect of YKSCH on TD-induced decreased Glu uptake was shown to occur through GLAST but not GLT-1 (Figure 4), a finding very similar to that for YKS in our previous report [13]. The difference of the effects of YKS and YKSCH is presumed to be due to the difference in the expression levels of protein and mRNA of GLAST, which should be clarified in the future study.

Alternatively, the amount of an active ingredient may be less or an inhibiting ingredient greater in YKSCH than in YKS. This possibility arises because the extraction efficiency of an ingredient from an herb is sometimes influenced by other herbs during the extraction process used to manufacture Kampo formulas. For example, the component that improves Glu uptake on TD astrocytes is reported to be glycyrrhizin and its metabolite glycyrrhetinic acid [14]. The glycyrrhizin content in the decoction of Glycyrrhiza with PT is lower than that of Glycyrrhiza alone [33, 34]. Thus, the glycyrrhizin content may be lower in YKSCH than YKS, thereby weakening the effect of YKSCH. The Japanese Pharmacopoeia 17th edition, supplement I, states that the daily dose of YKS (3.25 g of dried extract) contains 10–30 mg glycyrrhizin. Thus, the maximum concentration of glycyrrhizin in culture medium containing 100 *μ*g/mL YKS is 1122 nM. Considering our previous study showing the dose-response relationship of glycyrrhizin on Glu uptake by TD astrocytes [14], this maximum concentration of glycyrrhizin is the lower limit of the concentration showing improvement in TD-induced decreased Glu uptake by astrocytes. Although the difference in glycyrrhizin content between YKS and YKSCH might partially contribute to their differing efficacies with respect to Glu uptake, glycyrrhizin content alone cannot explain this difference. Considering that YKS components showing improving effects on Glu uptake are found not only in Glycyrrhiza but also in Bupleurum root, Poria sclerotium, Japanese Angelica root, and Cnidium rhizome [14], the difference in Glu uptake efficacy between YKS and YKSCH may result from differences in additive and/or synergic effects of their constituent plants.

We previously demonstrated that YKS inhibited Glu-induced cell death of primary cultured neuron [48] and PC12 cells [49], indicating that YKS has a protective effect against Glu neurotoxicity as one of the mechanisms underlying its neuropsychopharmacological effects [50]. In the present study, the inhibitory effects of YKSCH on Glu-induced PC12 cell death were examined, and its efficacy was compared to that of YKS. As shown in Figure 5, the efficacy of these two drugs is similar, suggesting that the YKS components in the YKSCH formula are responsible for the inhibitory effects of YKSCH. However, our data do not rule out the involvement of other additional herbs, because the neuroprotective effect has been found in CUP alone (Figure 6), as confirmed by previous reports [51, 52].

Several studies suggest that PC12 cells do not express a normal profile of NMDA receptors and that Glu cytotoxicity is mediated by the oxidative glutamatergic toxicity pathway, which is related to the system Xc^−^ [53–58]. The competition by Glu for the system Xc^−^ induces an imbalance in the homeostasis of cysteine, the precursor of glutathione. Thus, the inhibition of cystine uptake by high Glu concentrations may give rise to the inability to maintain intracellular glutathione levels needed to protect against oxidative injury [56]. We demonstrated previously that YKS protects against Glu cytotoxicity by augmenting gene expression of the system Xc^−^ subunits 4F2hc and xCT [15]. YKSCH is thought to exert its effects through the same mechanism as YKS.

## 5. Conclusion

We provide evidence that the pharmacological actions of YKS and YKSCH differ in some respects but are similar in others. YKSCH is less effective than YKS at ameliorating TD-induced decreases in Glu uptake by astrocytes, while YKSCH has similar inhibitory effects to YKS on Glu-induced PC12 cell death. These findings might be related to the observation that the antiaggressive effect of YKSCH is sometimes weaker than that of YKS. Additional studies are required to clarify the mechanisms underlying the differences in efficacy between these drugs and the involvement of PT and CUP in YKSCH.

## Figures and Tables

**Table 1:** Classification of the compounds identified in three-dimensional chromatogram.

Constituent herbs	Compounds	
	Yokukansan	Yokukansankachimpihange
Atractylodes Lancea	4E,6E,12E-tetradecatriene-8,10-diyne-1,3,14-	acetylatractylodin, atractylodin, atractylodinol
rhizome	triol, 12-isovaleroyl-2E,8E,10E- triene-4,6-	
	diyne-1,14-diol, 14-isovaleroyl-2E,8E,10E-	
	triene-4,6-diyne-1,12-diol, acetylatractylodin,	
	atractylodin, atractylodinol	
Cnidium rhizome	ferulic acid, ligustilide	ferulic acid, ligustilide
Uncaria hook	geissoschizine methyl ether, hirsuteine,	geissoschizine-O-methyl ether, hirsuteine,
	hirsutine	hirsutine
Japanese Angelica root	ligustilide, xanthotoxin	bergapten, ligustilide, xanthotoxin
Bupleurum root	saikosaponin b1, saikosaponin b2	saikosaponin b1, saikosaponin b2
Glycyrrhiza	formononetin, formononetin-7-*O*-glucoside,	formononetin, glycycoumarin, glycyroside,
	glycycoumarin, glycyroside, glycyrrhizin,	glycyrrhizin, isoliquiritigenin, isoliquiritin,
	isoliquiritigenin, isoliquiritin, isoliquiritin	isoliquiritin apioside, liquiritigenin, liquiritin,
	apioside, liquiritigenin, liquiritin, isoliquiritin	isoliquiritin apioside,
	apioside,	
Pinellia tuber		PT-H-15, PT-H-16
Citrus unshiu peel		hesperidin, narirutin

## Data Availability

The data used to support the findings of this study are available from the corresponding author upon request and permission.

## Supplementary Materials

Supplementary Materials Supplementary Figure 1: cultured rat cortical astrocytes. Astrocytes were fixed with 4% paraformaldehyde, stained with monoclonal antibodies for GFAP (brown), a specific marker for the astrocyte, and counterstained with hematoxylin (blue). Scale bar = 100 *μ*m.
