# Supplementary figures and images for: Comparison of the Effects of Yokukansan and Yokukansankachimpihange on Glutamate Uptake by Cultured Astrocytes and Glutamate-Induced Excitotoxicity in Cultured PC12 Cells
Source: Evid Based Complement Alternat Med. 2019 May 27;2019:9139536. doi: 10.1155/2019/9139536 (PMC6556795; doi:10.1155/2019/9139536)

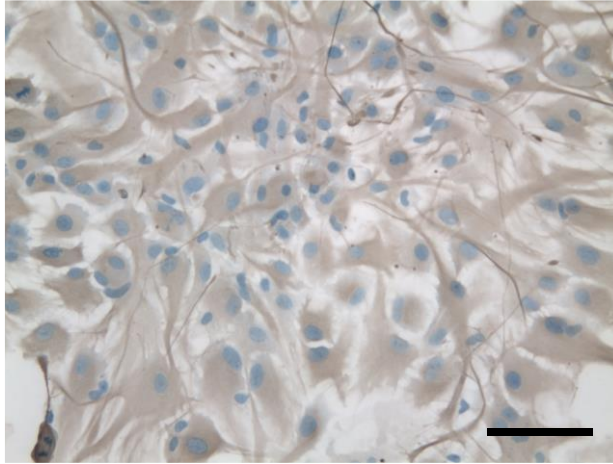

Supplementary Figure 1.

Supplement: Supplementary Materials — Supplementary Figure 1: cultured rat cortical astrocytes. Astrocytes were fixed with 4% paraformaldehyde, stained with monoclonal antibodies for GFAP (brown), a specific marker for the astrocyte, and counterstained with hematoxylin (blue). Scale bar = 100 μm. [file 9139536.f1.pdf]
